# Genetic association of *microRNA-146a* polymorphisms with the severity of coronary artery lesions in acute myocardial infarction

**DOI:** 10.1371/journal.pone.0339345

**Published:** 2025-12-26

**Authors:** Toan Hoang Ngo, Chuong Quoc Ho, Son Kim Tran

**Affiliations:** 1 Department of Internal Medicine, Faculty of Medicine, Can Tho University of Medicine and Pharmacy, Can Tho, Vietnam; 2 Center for Molecular Biomedicine, University of Medicine and Pharmacy, Ho Chi Minh, Vietnam; Scuola Superiore Sant'Anna, ITALY

## Abstract

**Background:**

Polymorphisms situated within the *microRNA-146a* gene have been extensively reported to fulfill a crucial regulatory function in controlling inflammatory responses and modulating gene expression. More recently, these specific genetic variants have also been implicated in the pathogenesis of coronary artery lesions; nevertheless, the current body of available evidence remains notably sparse.

**Objectives:**

To investigate the characteristics of the *microRNA-146a* gene polymorphisms rs2431697, rs57095329, and rs2910164, and their association with the severity of coronary artery lesions.

**Materials and methods:**

This comparative cross-sectional study included patients with acute myocardial infarction (AMI) and a control group, recruited from two hospitals in Can Tho City, Vietnam, between October 2023 and May 2025. All participants underwent clinical evaluation and coronary angiography. Subsequently, the *microRNA-146a* gene polymorphisms-rs2431697, rs57095329, and rs2910164-were analyzed using gene sequencing.

**Results:**

A total of 249 patients were included in the AMI group and 249 in the control group. The mean age of the patient cohort (66.65 ± 10.89 years) was similar to that of the controls (66.67 ± 14.12 years). Analysis of the rs2910164 polymorphism showed a lower frequency of the GG genotype in the AMI group compared with the control group (14.9% vs. 20.9%, p < 0.05), and all three SNPs met Hardy–Weinberg equilibrium criteria. Among patients with AMI, 30.5% had three-vessel coronary artery disease, and severe stenosis (≥90% luminal narrowing) was present in 78.3% of cases. Multivariate analysis demonstrated that the rs2431697 CC + TC genotype (OR = 0.10), the rs2910164 GG + CG genotype (OR = 3.23), diabetes mellitus (OR = 5.14), dyslipidemia (OR = 4.01), smoking (OR = 5.16), NT-proBNP ≥ 300 pg/mL (OR = 8.69), a GRACE score > 140 (OR = 10.82), and a TIMI score > 4 (OR = 6.50) were independent predictors of severe coronary stenosis (p < 0.05).

**Conclusion:**

Patients with acute myocardial infarction had a lower prevalence of the GG genotype of the rs2910164 polymorphism compared with the control group. The rs2910164 polymorphism of *microRNA-146a*-particularly genotypes carrying the G allele-along with a history of diabetes mellitus, dyslipidemia, smoking, NT-proBNP ≥ 300 pg/mL, a GRACE score > 140, and a TIMI score > 4 were identified as independent predictors of severe coronary artery stenosis. In contrast, the rs2431697 polymorphism with genotypes carrying the C allele was found to be a protective factor.

## 1. Introduction

Coronary artery disease (CAD) remains the leading cause of mortality worldwide [1], and acute myocardial infarction (AMI) represents its most severe clinical manifestation. The incidence of AMI is approximately 3.8% in individuals younger than 60 years and increases to 9.5% in those aged 60 years and older [2]. Notably, the number of younger patients with AMI has risen in recent years; ST-segment elevation myocardial infarction (STEMI) accounts for 12.8% of cases in patients under 45, with men representing nearly all affected individuals (96.8%) [3]. Despite advances in epidemiology, a considerable proportion of patients develop AMI at an early age without traditional cardiovascular risk factors, underscoring an important gap in current knowledge. Progress in genetic sequencing has opened new possibilities, suggesting that specific gene polymorphisms may contribute to this phenomenon [4].The role of microRNAs became clearer after Victor Ambros and Gary Ruvkun, in 1993, described a small RNA molecule capable of regulating gene expression by binding to the 3′UTR of its target mRNA, such as LIN-14. The term “microRNA” was later introduced by Tom Tuschl and David Bartel to designate this family of regulatory molecules [5]. MicroRNAs act within the RNA-induced silencing complex (RISC), leading to either mRNA degradation or inhibition of translation [6]. They participate in a wide range of biological processes, including development, differentiation, metabolism, and cellular stress responses. More than 2,000 microRNAs have been identified to date, and they are believed to influence roughly 30% of human gene activity [7]. Among these, *microRNA-146a* plays a key role in post-transcriptional gene regulation, particularly in inflammation through the IL-1R/TLR–NF-κB and JAK–STAT pathways, as well as in fibrotic processes. Because it participates in several central inflammatory signaling pathways, altered *microRNA-146a* function has attracted increasing interest regarding its potential contribution to disease mechanisms [8]. Polymorphisms in the *microRNA-146a* gene have been associated with a variety of disorders. The rs2910164 variant, in particular, has been shown to modify gene expression and is linked to several inflammatory conditions [9,10]. More recent reports suggest that this polymorphism also affects circulating inflammatory markers, and that the GG genotype may increase the risk of more severe coronary artery lesions in patients with acute coronary syndrome [11]. In Vietnam, evidence on this topic remains limited. Therefore, the present study was conducted to characterize microRNA-146a gene polymorphisms and assess their association with the severity of coronary artery lesions.

## 2. Materials and methods

### 2.1. Study design and population

This comparative cross-sectional study was conducted on patients admitted to the Department of Cardiology and the Interventional Cardiology Unit of Can Tho Central General Hospital, as well as the Cardiology Department of Can Tho University of Medicine and Pharmacy Hospital, from October 2023 to May 2025. Inclusion and exclusion criteria were applied to ensure the appropriate selection of study participants. The disease group consisted of patients diagnosed with acute myocardial infarction according to the criteria of the European Society of Cardiology [12], while the control group included individuals without acute myocardial infarction who were matched to the cases by age and sampling location (recruited from the same clinical departments). Exclusion criteria for the disease group included contraindications to percutaneous coronary angiography, psychiatric disorders or dementia that prevented reliable interviewing, and chronic kidney disease with an estimated glomerular filtration rate below 30 mL/min/1.73 m². At the end of the recruitment period, 249 patients met the criteria for the AMI group, and an equal number of matched controls (n = 249) were enrolled in the study.

### 2.2. Data collection

All participants were assessed for anthropometric characteristics, including age, sex, and body mass index. Clinical evaluations were performed by cardiologists to document baseline comorbidities such as hypertension [13], diabetes mellitus [14], dyslipidemia [15], prior stroke, family history of cardiovascular disease, smoking status, and sedentary lifestyle. In patients with acute myocardial infarction, high-sensitivity C-reactive protein (hs-CRP) and high-sensitivity troponin T levels were measured, and risk stratification was carried out using the GRACE and TIMI scores, along with clinical severity assessment based on the Killip classification [16]. Coronary angiography was subsequently performed in the AMI group to identify the pattern and extent of coronary artery involvement. Three-vessel disease was defined as significant stenosis (≥50%) in all three major coronary arteries, whereas stenosis of ≥90% was classified as severe. The prevalence and characteristics of coronary lesions were recorded accordingly [17].

### 2.3. Genotyping

A 2 mL peripheral blood sample was collected into an EDTA anticoagulant tube at hospital admission, concurrently with the high-sensitivity troponin T (hs-TnT) test. Genomic DNA was extracted from whole blood using the GeneJET Genomic DNA Purification Kit (Thermo Scientific, USA). The extracted DNA was stored at −30°C until analysis. PCR primers and sequencing strategies were designed to analyze three single nucleotide polymorphisms (SNPs) of the microRNA-146a gene: rs2431697, rs57095329, and rs2910164. Reference gene sequences for microRNA-146a were obtained from the NCBI database (accession number NC_000005.10). Target regions were amplified using primers synthesized by Integrated DNA Technologies (IDT), USA. Each PCR reaction had a total volume of 15 μL, containing 25–50 ng of genomic DNA, 0.5 U of Taq Hot Start Polymerase, 0.1 μM of each primer, 200 μM of each dNTP, and 1X PCR buffer. Amplifications were performed using the SimpliAmp Thermal Cycler (Thermo Scientific), with an annealing temperature of 58°C. PCR products were analyzed on 1.5% agarose gel electrophoresis, then purified using ExoSAP-IT reagent (Thermo Scientific) to remove residual primers and nucleotides. Direct sequencing of the PCR products was performed using the Sanger method with the BigDye™ Terminator v3.1 Cycle Sequencing Kit (Applied Biosystems). Sequencing reactions were analyzed using the ABI 3500 Genetic Analyzer (Applied Biosystems). SNPs were identified and genotyped using CLC Main Workbench v5.5 software. The resulting data were compiled and subjected to statistical analysis (S1 Table in S1 File).

### 2.4. Statistical analysis

Data were analyzed using SPSS version 26.0. Categorical variables were expressed as frequencies and percentages. Continuous variables were summarized as mean ± standard deviation for normally distributed data, and as median with interquartile range for non-normally distributed data. Differences in categorical variables were assessed using the Chi-square test or Fisher’s exact test when appropriate. Comparisons of continuous variables between two groups were performed using the Student’s t-test. Logistic regression analysis was conducted to identify factors independently associated with the outcomes of interest.

### 2.5. Ethical approval

All participants were clearly informed about the study and provided written informed consent prior to enrollment. They were also allowed to withdraw from the study at any stage without any consequences. All data collected were used solely for research purposes and were not disclosed without the participants’ explicit consent. The costs of genetic sequencing and any other expenses incurred during the study were fully covered by the research team. The study was conducted after receiving approval from the Ethics Committee of Can Tho University of Medicine and Pharmacy, and it complied with the ethical principles of the Declaration of Helsinki. The study was approved by the Ethics Committee in biomedical research of Can Tho Univeristy of Medicine and Pharmacy with the Decision No. 23.009.NCS/PCT-HDDD on June 15, 2023.

## 3. Results

### 3.1. Characteristics of the rs2431697, rs57095329, and rs2910164 *microRNA-146a* polymorphisms

As shown in Table 1, the mean age in the AMI group was 66.65 ± 10.89 years, which was comparable to that of the control group (66.67 ± 14.12 years). The proportion of male patients was higher in the AMI group than in the controls (69.1% vs. 43.0%). Similarly, dyslipidemia was more frequent among AMI patients (75.5% vs. 10.0%; p < 0.05), and the proportion of current smokers was also higher in the AMI group (48.6% vs. 29.7%; p < 0.05).

**Table 1:** Baseline characteristics of the study population.

Factors	AMI (n = 249)	Control (n = 249)	Clinical presentations of acute myocardial infarction		p^1^	p^2^
			STEMI (n = 138)	NSTEMI (n = 111)		
Age (years)	66.65 ± 10.89	66.67 ± 14.12	66.91 ± 10.36	66.33 ± 11.55	0.986	0.677
Male	172 (69.1)	107 (43.0)	98 (71.0)	74 (66.7)	<0.001	0.461
Body mass index (Kg/m²)	21.77 ± 2.31	21.97 ± 3.22	21.61 ± 2.20	21.97 ± 2.43	0.435	0.230
Hypertension	181 (72.7)	170 (68.3)	102 (73.9)	79 (71.2)	0.280	0.629
Diabetes mellitus	54 (21.7)	34 (13.7)	28 (20.3)	26 (23.4)	0.019	0.551
Dyslipidemia	170 (68.3)	64 (25.7)	97 (70.3)	73 (65.8)	<0.001	0.446
Family history of coronary artery disease	48 (19.3)	34 (13.7)	30 (21.7)	18 (16.2)	0.091	0.272
History of stroke	17 (6.8)	13 (5.2)	9 (6.5)	8 (7.2)	0.458	0.831
Smoking	121 (48.6)	74 (29.7)	68 (49.3)	53 (47.7)	<0.001	0.811
Sedentary lifestyle	75 (30.1)	63 (25.3)	40 (29.0)	35 (31.5)	0.230	0.663
Killip classification	1.39 ± 0.81	–	1.41 ± 0.79	1.37 ± 0.83	–	0.672
GRACE score	116.32 ± 33.25	–	119.81 ± 30.80	111.97 ± 35.74	–	0.064
TIMI score	3.99 ± 1.24	–	4.31 ± 1.11	3.59 ± 1.27	–	<0.001
CRPhs (mg/L)	4.34 (1.41-13.90)	–	6.00 (1.60-16.25)	2.72 (1.28-9.37)	–	0.009
Troponin Ths (ng/mL)	1.39 (0.36-4.79)	–	1.60 (0.47-5.40)	1.30 (0.30-4.41)	–	0.303
NT-proBNP (pg/mL)	866.60 (258.40-2223.75)	–	1240.05 (494.40-2510.32)	408.40 (184.20-1882.20)	–	<0.001

p^1^: cases vs controls; p^2^: STEMI vs NSTEMI.

The analysis showed that patients in the case group had a lower frequency of the GG genotype of rs2910164 compared with the control group (14.9% vs. 20.9%, p < 0.05). However, no significant differences were observed in the genotype distributions of rs2431697 and rs57095329 between the two groups (p > 0.05). All three SNPs were in Hardy-Weinberg equilibrium (Table 2).

**Table 2:** Characteristics of the rs2431697, rs57095329, and rs2910164 *microRNA-146a* polymorphisms.

Characteristics	AMI (n = 249)	Control (n = 249)	p
rs2431697			
CC genotype	03 (1.2)	06 (2.4)	0.099
TC genotype	33 (13.3)	48 (19.3)	
TT genotype	213 (85.5)	195 (78.3)	
HWE Chi-squared (χ²)	1.672	2.035	–
HWE p-value	0.196	0.154	
rs57095329			
GG genotype	16 (6.4)	18 (7.3)	0.880
AG genotype	83 (33.3)	86 (34.5)	
AA genotype	150 (60.3)	145 (58.2)	
HWE Chi-squared (χ²)	0.943	1.097	–
HWE p-value	0.331	0.295	
rs2910164			
GG genotype	37 (14.9)	52 (20.9)	0.039
CG genotype	111 (44.6)	121 (48.6)	
CC genotype	101 (40.5)	76 (30.5)	
HWE Chi-squared (χ²)	0.512	0.090	–
HWE p-value	0.474	0.764	

No significant differences in the distribution of rs2431697, rs57095329, or rs2910164 genotypes were observed according to the presence of hypertension, diabetes mellitus, or dyslipidemia. Patients carrying the GG + CG genotypes of rs2910164 had a higher mean Killip class compared with those carrying the CC genotype (1.52 ± 0.92 vs. 1.21 ± 0.55, p < 0.05) (Table 3).

**Table 3:** Distribution of microRNA-146a polymorphisms according to clinical Risk factors in patients with acute myocardial infarction.

Characteristics	rs2431697		rs57095329		rs2910164		p^1^	p^2^	p^3^
	CC+TC	TT	GG+AG	AA	GG+CG	CC			
Hypertension	25 (69.4)	156 (73.2)	73 (73.7)	108 (72.0)	111 (75.0)	70 (69.3)	0.636	0.763	0.322
Diabetes mellitus	07 (19.4)	47 (22.1)	20 (20.2)	34 (22.7)	38 (25.7)	16 (15.8)	0.724	0.644	0.064
Dyslipidemia	23 (63.9)	147 (69.0)	71 (71.7)	99 (66.0)	104 (70.3)	66 (65.3)	0.541	0.343	0.412
Family history of coronary artery disease	06 (16.7)	42 (19.7)	16 (16.2)	32 (21.3)	30 (20.3)	18 (17.8)	0.668	0.311	0.631
History of stroke	02 (5.6)	15 (7.0)	06 (6.1)	11 (7.3)	13 (8.8)	04 (4.0)	1.0	0.697	0.138
Smoking	16 (44.4)	105 (49.3)	50 (50.5)	71 (47.3)	82 (55.4)	39 (38.6)	0.590	0.624	0.009
Killip classification	1.39 ± 0.73	1.39 ± 0.82	1.46 ± 0.91	1.35 ± 0.73	1.52 ± 0.92	1.21 ± 0.55	0.970	0.260	<0.001
GRACE score	119.44 ± 35.87	115.79 ± 32.85	117.58 ± 36.05	115.49 ± 31.37	119.86 ± 36.88	111.13 ± 26.41	0.543	0.629	0.031
TIMI score	4.17 ± 1.38	3.96 ± 1.21	4.07 ± 1.26	3.93 ± 1.22	4.11 ± 1.29	3.81 ± 1.15	0.351	0.393	0.064

### 3.2. Association between the polymorphisms of the *microRNA-146a* gene and factors with the severity of coronary artery lesions

**Fig 1:**
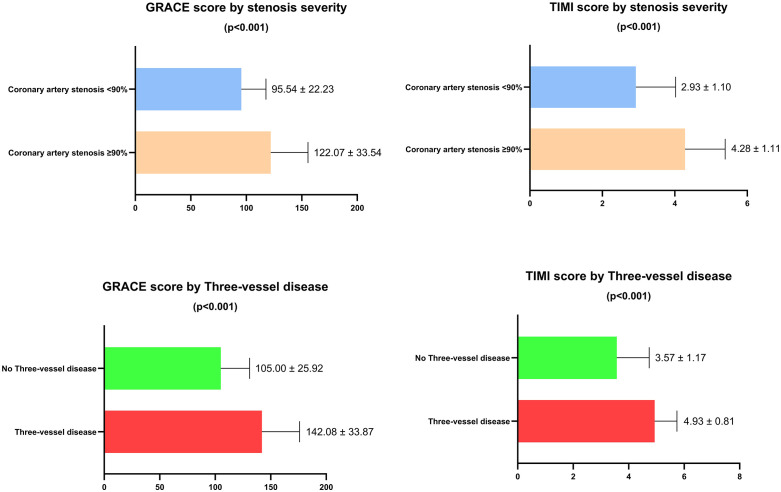
Comparison of GRACE and TIMI scores according to the severity of coronary artery stenosis and the presence of triple-vessel disease.

Among the patients with acute myocardial infarction included in the study, the prevalence of triple-vessel disease was 30.5%, and severe coronary stenosis (≥90%) was observed in 78.3%. Patients with STEMI exhibited a higher proportion of ≥90% coronary stenosis compared with those with NSTEMI (86.2% vs. 68.5%; p < 0.05) (Table 4). Patients with ≥90% coronary stenosis had a lower frequency of the CC + TC genotypes of rs2431697 compared with those with stenosis <90% (11.3% vs. 25.9%). In contrast, the proportions of the GG + AG genotypes of rs57095329 (43.1% vs. 27.8%) and the GG + CG genotypes of rs2910164 (63.1% vs. 46.3%) were higher in the ≥ 90% stenosis group than in the < 90% group (p < 0.05) (Table 5). When comparing GRACE and TIMI scores according to the extent of coronary artery involvement, patients with severe coronary stenosis and those with triple-vessel disease exhibited higher mean GRACE and TIMI scores (p < 0.05) (Fig 1).

**Table 4:** Characteristics of coronary artery lesions.

Characteristics of coronary artery lesions	Total (n = 249)	Clinical presentations of acute myocardial infarction		p
		STEMI	NSTEMI	
Right coronary artery (RCA) stenosis	136 (54.6)	87 (63.0)	49 (44.1)	0.003
Left anterior descending (LAD) artery stenosis	188 (75.5)	116 (84.1)	72 (64.9)	<0.001
Left circumflex (LCX) artery stenosis	131 (52.6)	67 (48.6)	64 (57.7)	0.153
Three-vessel coronary artery disease	76 (30.5)	41 (29.7)	35 (31.5)	0.756
Severe coronary artery stenosis (≥90%)	195 (78.3)	119 (86.2)	76 (68.5)	0.001

**Table 5:** *microRNA-146a* polymorphisms and coronary artery disease severity.

Factors	Severe coronary artery stenosis (≥90%)		Three-vessel coronary artery disease		p^1^	p^2^
	Yes (n = 195)	No (n = 54)	Yes (n = 76)	No (n = 173)		
rs2431697						
CC+TC	22 (11.3)	14 (25.9)	15 (19.7)	21 (12.1)	0.007	0.116
TT	173 (88.7)	40 (74.1)	61 (80.3)	152 (87.9)		
rs57095329						
GG+AG	84 (43.1)	15 (27.8)	25 (32.9)	74 (42.8)	0.042	0.142
AA	111 (56.9)	39 (72.2)	51 (67.1)	99 (57.2)		
rs2910164						
GG+CG	123 (63.1)	25 (46.3)	53 (69.7)	95 (54.9)	0.026	0.028
CC	72 (36.9)	29 (53.7)	23 (30.3)	78 (45.1)		
Risk factor						
Hypertension	151 (77.4)	30 (55.6)	69 (90.8)	112 (64.7)	0.001	<0.001
Diabetes mellitus	52 (26.7)	02 (3.7)	39 (51.3)	15 (8.7)	<0.001	<0.001
Dyslipidemia	144 (73.8)	26 (48.1)	60 (78.9)	110 (63.6)	<0.001	0.016
Smoking	108 (55.4)	13 (24.1)	45 (59.2)	76 (43.9)	<0.001	0.026
Killip classification	1.49 ± 0.88	1.04 ± 0.19	2.00 ± 0.98	1.13 ± 0.53	<0.001	<0.001

In the multivariable analysis (Table 6), the following factors were identified as independent predictors of severe coronary stenosis: the CC + TC genotypes of rs2431697 (OR=0.10), the GG + CG genotypes of rs2910164 (OR=3.23), a history of diabetes mellitus (OR=5.14), dyslipidemia (OR=4.01), smoking (OR=5.16), NT-proBNP ≥ 300 pg/mL (OR=8.69), GRACE score >140 (OR=10.82), and TIMI score >4 (OR=6.50) (p < 0.05). Regarding factors associated with triple-vessel coronary artery disease (Table 7), the multivariable regression analysis identified diabetes mellitus (OR=10.97), a TIMI score >4 (OR=4.90), and Killip class ≥2 (OR=14.10) as independent predictors of triple-vessel disease (p < 0.05).

**Table 6:** Univariate and multivariate regression analysis of risk factors associated with the severity of coronary artery stenosis.

Severe coronary artery stenosis (≥90%)	Univariate logistic regression		Multivariate logistic regression	
	OR (95% CI)	p	OR (95% CI)	p
rs2431697 CC+TC	0.36 (0.17–0.77)	0.008	0.10 (0.02–0.40)	0.001
rs57095329 GG+AG	1.97 (1.02–3.80)	0.044	1.86 (0.65–5.33)	0.246
rs2910164 GG+CG	1.98 (1.08–3.64)	0.028	3.23 (1.14–9.19)	0.028
Age	1.01 (0.98–1.04)	0.354	–	–
Hypertension	2.74 (1.46–5.17)	0.002	1.15 (0.47–2.80)	0.760
Diabetes mellitus	9.45 (2.22–40.21)	0.002	5.14 (1.03–25.54)	0.046
Dyslipidemia	3.04 (1.63–5.66)	<0.001	4.01 (1.65–9.70)	0.002
Smoking	3.91 (1.97–7.76)	<0.001	5.16 (2.00–13.34)	<0.001
NTproBNP ≥ 300 pg/mL	6.42 (3.35–12.33)	<0.001	8.69 (3.22–23.41)	<0.001
CRPhs ≥ 10 mg/L	3.84 (1.65–8.96)	0.002	1.03 (0.32–3.30)	0.954
GRACE score >140	23.56 (3.18–174.33)	0.002	10.82 (1.11–105.13)	0.040
TIMI score >4	7.76 (3.17–18.96)	<0.001	6.50 (1.91–22.10)	0.003
Killip ≥2	11.01 (2.59–46.70)	0.001	1.24 (0.20–7.60)	0.816

**Table 7:** Univariate regression analysis of risk factors associated with triple-vessel coronary artery disease.

Three-vessel coronary artery disease	Univariate logistic regression		Multivariate logistic regression	
	OR (95% CI)	p	OR (95% CI)	p
rs2431697 CC+TC	1.78 (0.86–3.68)	0.120	–	–
rs57095329 GG+AG	0.66 (0.37–1.15)	0.144	–	–
rs2910164 GG+CG	1.89 (1.07–3.36)	0.029	1.01 (0.43–2.33)	0.991
Age	0.98 (0.96–1.01)	0.229	–	–
Hypertension	5.37 (2.32–12.41)	<0.001	2.77 (0.88–8.73)	0.083
Diabetes mellitus	11.10 (5.54–22.24)	<0.001	10.97 (3.87–31.11)	<0.001
Dyslipidemia	2.15 (1.14–4.04)	0.018	1.31 (0.52–3.29)	0.566
Smoking	1.85 (1.07–3.20)	0.027	1.50 (0.64–3.52)	0.354
NTproBNP ≥ 300 pg/mL	8.13 (3.12–21.20)	<0.001	2.79 (0.82–9.44)	0.099
CRPhs ≥ 10 mg/L	6.76 (3.71–12.30)	<0.001	1.37 (0.51–3.64)	0.528
GRACE score >140	10.01 (5.19–19.31)	<0.001	1.23 (0.38–3.95)	0.726
TIMI score >4	9.45 (5.03–17.73)	<0.001	4.90 (2.01–11.94)	<0.001
Killip ≥2	26.73 (12.37–57.75)	<0.001	14.10 (4.20–47.40)	<0.001

## 4. Discussion

The frequency of the GG genotype of rs2910164 was lower in the case group than in the controls (14.9% vs. 20.9%). All three SNPs-rs2431697, rs57095329, and rs2910164-were in Hardy-Weinberg equilibrium. Multivariable analysis further demonstrated that the CC+TC genotypes of rs2431697, the GG+CG genotypes of rs2910164, a history of diabetes mellitus, dyslipidemia, smoking, NT-proBNP ≥ 300 pg/mL, GRACE score >140, and TIMI score >4 were independent predictors of severe coronary stenosis.

Our study was designed to evaluate the characteristics of the *microRNA-146a* polymorphisms rs2431697, rs57095329, and rs2910164 and their associations with coronary artery lesions, based on clearly defined inclusion and exclusion criteria. Genotyping of all three polymorphisms was performed using sequencing in a modern molecular biology laboratory, following a transparent and reproducible workflow. Genotype distributions were comprehensively characterized, compared with those of a control group, and analyzed to identify the associations of rs2910164 and rs2431697 with the severity of coronary artery stenosis. However, this study has certain limitations. It was conducted at a single center in Can Tho, Vietnam, where clinical and laboratory characteristics may differ from those of other populations, potentially affecting the generalizability of the findings. Therefore, larger, multicenter studies are recommended to further elucidate the associations between the rs2431697, rs57095329, and rs2910164 *microRNA-146a* polymorphisms and coronary artery lesions in patients with acute myocardial infarction.

Several studies have investigated the association between *microRNA-146a* gene polymorphisms and coronary artery disease. According to Yaqin Wang et al., in an analysis of 353 patients with CAD and 368 controls, the genotype frequencies of rs2431697 and rs2910164 differed significantly between the two groups. The T allele of rs2431697 and the C allele of rs2910164 were more common among CAD patients. Carriers of the T allele of rs2431697 had a higher risk of CAD, whereas the G allele of rs2910164 was associated with a significantly reduced risk. However, neither polymorphism correlated with the severity of coronary lesions [18]. Reyes-García et al. reported that rs2431697 did not increase the risk of acute coronary syndrome onset, although T-allele carriers exhibited significantly higher levels of neutrophil extracellular traps (NETs). This polymorphism has been linked to subclinical inflammation and a higher incidence of adverse outcomes [19]. Current evidence on rs57095329 in CAD remains limited. This polymorphism has been associated with inflammatory and metabolic disorders, and the AG and GG genotypes, as well as the G allele, have been linked to an increased risk of type 1 diabetes among Egyptian patients [20]. In our study, we found that rs2910164 carriers of the G allele had an independently increased risk of severe coronary stenosis (≥90%), whereas rs2431697 carriers of the C allele appeared to be protected. Similar to our findings, Hao Qiu et al. reported that the GG genotype (10.20% vs. 13.92%) and the G allele (34.01% vs. 37.25%) were less frequent in myocardial infarction patients compared with controls [21]. Rashid Mir et al. also demonstrated that the GG genotype and G allele of rs2910164 were associated with a higher risk of coronary artery disease [22]. Data on the relationship between rs2910164 and CAD risk remain inconsistent. A meta-analysis by Qinxue Bao et al., including 18 studies with 6,859 patients and 8,469 controls, showed that the G allele of rs2910164 was associated with a significantly reduced risk of CAD in the allelic model (OR = 0.86), homozygous model (OR = 0.79), and heterozygous model (OR = 0.89) in the overall population. In subgroup analyses, carriers of the G allele and the GG genotype had a lower risk of CAD in Chinese populations [23]. Conversely, Xiang-Rui Qiao et al. reported that patients with acute coronary syndrome had a higher frequency of the GG genotype compared with controls (17.61% vs. 13.23%), conferring an increased risk of disease (OR = 1.551; 95% CI: 1.090–2.205; p < 0.05). Moreover, the GG genotype was associated with more severe coronary lesions, with mean Gensini scores of 52.46 ± 2.11, 63.31 ± 2.35, and 84.89 ± 4.00 in patients with the CC, CG, and GG genotypes, respectively [11]. Previous studies have suggested that rs2910164 may alter the expression of *microRNA-146a*, thereby modifying inflammatory responses [24]. In healthy individuals, carriers of the GG genotype exhibit slightly higher serum TNF-α levels compared with those carrying CC or CG genotypes, although IL-1β and IL-6 levels do not differ significantly. Interestingly, among patients with acute coronary syndrome, all three inflammatory markers were significantly higher in individuals with the CG and GG genotypes compared with CC carriers, and TNF-α levels were greatest in those with the GG genotype [11]. These findings collectively suggest that the association between rs2910164 and coronary artery lesions may be mediated indirectly through altered *microRNA-146a* expression and corresponding inflammatory responses [11,23,24].

In addition to their association with the severity of coronary artery lesions, *microRNA-146a* polymorphisms have also been reported to influence gene expression. Krystian Jazdzewski and colleagues demonstrated that the rs2910164 C allele reduces *microRNA-146a* expression; specifically, individuals carrying the CC genotype exhibited a 3.9-fold lower expression level compared with those with the GG genotype [25]. Similarly, Xinling Zhang and co-workers reported that the C allele of rs2910164 decreases *microRNA-146a* expression but increases the expression of NOS1. Notably, a sequence within the 3′-untranslated region of NOS1, together with luciferase reporter assays, confirmed that NOS1 is a direct target gene of *microRNA-146a*. Their study further showed that both mRNA and protein levels of NOS1 were markedly reduced in U251 cells treated with *microRNA-146a* mimics compared with scramble controls, whereas cells treated with *microRNA-146a* inhibitors exhibited increased NOS1 expression [26]. Consistent findings were reported by Jian Ding and colleagues, who assessed the effect of rs2910164 on *microRNA-146a* and NOS1 expression. By treating PC-3 cells with *microRNA-146a* mimics or inhibitors to validate the negative regulatory relationship between *microRNA-146a* and NOS1, they employed real-time PCR and Western blot analyses to quantify NOS1 mRNA and *microRNA-146a* expression. Their results demonstrated that NOS1 mRNA and protein expression levels were significantly reduced in PC-3 cells treated with *microRNA-146a* mimics or NOS1 siRNA compared with random controls, whereas treatment with *microRNA-146a* inhibitors led to increased NOS1 expression. These findings further reinforce the reciprocal regulatory interaction between microRNA-146a and NOS1 [27]. Moreover, NOS1 has been shown to play a role in endothelial function and has been associated with coronary artery disease [28]. This suggests that *microRNA-146a* SNPs, through their regulatory influence on gene expression, may indirectly affect NOS1 and thereby potentially impact endothelial function. Although this represents a relatively novel finding, additional evidence is needed to substantiate this hypothesis [29].

## 5. Conclusion

Patients with acute myocardial infarction exhibited a lower frequency of the GG genotype of the rs2910164 *microRNA-146a* polymorphism compared with controls. The rs2910164 polymorphism-particularly genotypes carrying the G allele-together with a history of diabetes mellitus, dyslipidemia, smoking, NT-proBNP ≥ 300 pg/mL, a GRACE score >140, and a TIMI score >4, were identified as independent predictors of severe coronary stenosis. In contrast, rs2431697 genotypes carrying the C allele appeared to confer a protective effect.

## Supporting information

S1 File Single nucleotide polymorphism detection procedure of microRNA-146a. (DOCX)
