# Supplementary material for: Genetic association of microRNA-146a polymorphisms with the severity of coronary artery lesions in acute myocardial infarction
Source: PLoS One. 2025 Dec 26;20(12):e0339345. doi: 10.1371/journal.pone.0339345 (PMC12742729; doi:10.1371/journal.pone.0339345)
Supplement: S1 File — (DOCX) [file pone.0339345.s001.docx]

**APPENDIX**

**Single nucleotide polymorphism detection procedure of microRNA-146a**

*Sample collection and DNA extraction*

For patients, we collected 2 mL of peripheral blood with EDTA anticoagulant, then genomic DNA was extracted using the GeneJET Genomic DNA Purification Kit (Thermo Scientific, USA) and stored at −30°C.

*SNP analyses*

PCR and sequencing primers were designed for analyzing the SNPs of miR-164a gene (rs2431697, rs57095329, and rs2910164). The reference genomic sequences of miR-164a gene was obtained from the National Center for Biotechnology Information Consensus CDS database, with accession number NC_000005.10 (https://www.ncbi.nlm.nih.gov/projects/CCDS/CcdsBrowse.cgi).

The amplicons were amplified by primers synthesized by IDT (Integrated DNA Technologies), and listed in Table S1. PCRs (15 μl) contained 25-50 ng of genomic DNA, 0.5 U Taq Hot Start Polymerase (Takara Bio), 0.1 μM each forward and reverse primers, 200 μM of each dNTP, 1X PCR Buffer. The reactions were ran in SimpliAmp Thermal Cycler (Thermo Scientific) with the annealing temperature was set to 58^0^C. The PCR products were analyzed on 1.5% agarose gel electrophoresis and then were purified with the ExoSAP-IT reagent (Thermo Scientific). The amplicons were direct sequenced by Sanger method using Bigdye^TM^ Terminator v3.1 Cycle Sequencing Kit (Applied Biosystems) in both forward and reverse directions. The sequencing reactions were analyzed on an ABI 3500 Genetic Analyzer (Applied Biosystems). The sequencing results of SNPs were analyzed with CLC Mainworkbench v5.5 software.

**Table S1**: Primer sequences for PCR amplification

| **SNP** | **Primer name** | **Primer sequence (5’ – 3’)** | **Length (bp)** |
| --- | --- | --- | --- |
| rs2431697 | RNA-697F | GACCCACTTTGTCATACACG | 213 |
|  | RNA-697R | GAGGCAAGCCAATGAAGCAG |  |
| rs57095329 | RNA-329F | AGGTTTTGGCTGAAACTCAG | 191 |
|  | RNA-329R | AGAGCGTTCTGTGCAGGATG |  |
| rs2910164 | RNA-164F | AGGAAGCAGCTGCATTGGAT | 296 |
|  | RNA-164R | GCCTGAGACTCTGCCTTCTG |  |
