# Supplementary material for: Genetic association of microRNA-146a polymorphisms with the severity of coronary artery lesions in acute myocardial infarction
Source: PLoS One. 2025 Dec 26;20(12):e0339345. doi: 10.1371/journal.pone.0339345 (PMC12742729; doi:10.1371/journal.pone.0339345)
Supplement: Attachment — Submitted filename: Respond Reviewer.docx [file pone.0339345.s003.docx]

**Response to Reviewer 1 Comments**

Dear Reviewers, we are happy to receive your comments. Thank you for taking the time to read and comment on our manuscript titled ***Genetic variations of microRNA-146a and their association with the severity of coronary artery lesions in patients with acute myocardial infarction.*** We have prepared the manuscript according to the your comments.

Our responses are below:

**Major**

**Point 1:** The title is too long and has grammar errors, please check.

**Response 1:** We have revised the title accordingly: ***Genetic variations of microRNA-146a and their association with the severity of coronary artery lesions in patients with acute myocardial infarction***

**Point 2:** The introduction part is scanty. The authors should clarify the reasons why this gene was chosen to study, especially the connection between these polymorphisms and acute myocardial infarction. What are the roles of these SNPs in the development of acute myocardial infarction? Furthermore, the authors did not clearly present what the aims of this study are.

**Response 2:** We sincerely appreciate the reviewer’s insightful comments. We have added a description of the role of microRNA-146a to the Introduction and have also incorporated the study objectives into both the Abstract and the Introduction.

**Point 3:** The results were confused as the authors presented many comparisons. I suggest that the authors should clarify the aims of the study and focus on these aims while presenting the results.

**Response 3:** We have revised the multivariable analysis to place greater emphasis on evaluating the impact of risk factors and the rs2431697, rs57095329, and rs2910164 polymorphisms on the severity of coronary artery lesions. We sincerely appreciate your valuable feedback.

**Point 4:** In table 3, why did the authors combine 2 genotypes in 1 group? Any specific reasons for that?

**Response 4:** The combination of two genotypes in the analysis was intended to measure the effect of genotypes carrying the risk allele compared with those carrying no risk alleles. In addition, genotype-based analysis captures a full range of genetic inheritance models, including dominant, recessive, homozygous, and heterozygous patterns. While allele-based analysis only reflects differences in allele frequencies between cases and controls, genotype analysis allows for a direct evaluation of the genetic effect of a variant-for example, the dominant model (AA + Aa vs. aa), the recessive model (AA vs. Aa + aa), and assessment of Hardy-Weinberg equilibrium

**Point 5:** Suggest that the authors should focus on univariate and multivariate logistic regression analyses for AMI (with and without disease) and severity of coronary results.

**Response 5:** We sincerely appreciate the reviewer’s valuable comments. Our study aimed to evaluate the impact of microRNA-146a gene polymorphisms on the severity of coronary artery lesions in patients with acute myocardial infarction, rather than to assess their contribution to the risk of myocardial infarction onset. Nevertheless, we have further clarified both the univariable and multivariable models by incorporating clinical risk factors, biomarkers, and clinical scoring systems to better quantify their effects on the severity of coronary artery involvement.

**Response to Reviewer 2 Comments**

**Major**

**Point 1:** The manusript idea is new- Title is long but accepted. Subjects and Methods: are well described , however I prefer to measure the expression of Mir 146 beside genotypings. Results: is clear but I prefer to evaluate Hardy-Weinberg equilibrium in groups, Measurment the Expression of mir 146 in cases will give a view on the effect of polymorphisms on mir 146 production.

**Response 1:** We sincerely appreciate the reviewer’s insightful comments. Assessing the expression levels of microRNA-146a in addition to genotyping is indeed an excellent suggestion. However, due to limitations in our current resources, we were unable to perform this analysis in the present study. We will consider incorporating this important aspect in future research if feasible. The Hardy–Weinberg equilibrium assessment has now been added to the Results section of the revised manuscript.

**Response to Reviewer 3 Comments**

**Major**

**Point 1:** Please elaborate why miRNA-146a was chosen as the vascular miRNA among the numerous documented miRNA markers associated with CVD risk. The study conclusions would be - strengthened by measuring a larger panel of miRNAs in a larger cohort.

**Response 1:** We sincerely appreciate the reviewer’s valuable comments. Our study was conducted based on the premise that microRNA-146a is a circulating microRNA that plays a pivotal role in post-transcriptional gene regulation through its control of key inflammatory pathways, including the IL-1R/TLRs–NFκB axis, the Janus kinase (JAK)–STAT pathway, and fibrosis-related signaling. As microRNA-146a is involved in nearly all major regulatory pathways of the inflammatory response, alterations affecting this microRNA may influence inflammatory activity and thereby contribute to coronary artery injury. Supporting evidence shows that the rs2910164 polymorphism can alter gene expression, is associated with dysregulation of inflammatory cytokines, and correlates with the severity of coronary artery lesions

**Point 2:** Provide a more thorough description of the SNPs and their potential impact on miRNA expression, as this information is currently lacking from the manuscript.

**Response 2:** This represents a limitation of our study. Due to restricted resources, our investigation was limited to genotypic characterization and did not include an assessment of how these polymorphisms influence gene expression or circulating microRNA levels in plasma

**Point 3:** Please introduce the aim of the study in the introduction section.

**Response 3:** We have added the study objectives and expanded the Introduction accordingly

**Point 4:** The results section would be better if it were divided into subsections to make the message clearer to the reader. There are also too many tables; Tables 1 and 2 could be grouped together.

**Response 4:** Thank you very much for your comment. Table 2 is included in the subsection titled ‘Characteristics of the rs2431697, rs57095329, and rs2910164 microRNA-146a polymorphisms,’ which has been separated from the general characteristics section. The intention of this structure is to emphasize that this subsection focuses specifically on describing the genetic polymorphisms and evaluating their effects on clinical risk factors

**Minor**

**Point 5:** Include the missing p-values for the comparisons in both the abstract and the results section. The alleles and genotypes should be italicized.

**Response 5:** Thank you very much. We have incorporated the requested revisions into the manuscript.
