# Supplementary material for: Genetic association of microRNA-146a polymorphisms with the severity of coronary artery lesions in acute myocardial infarction
Source: PLoS One. 2025 Dec 26;20(12):e0339345. doi: 10.1371/journal.pone.0339345 (PMC12742729; doi:10.1371/journal.pone.0339345)
Supplement: Attachment — Submitted filename: Respond Editor.docx [file pone.0339345.s004.docx]

**Response to Editor Comments**

Dear Editor, we are happy to receive your comments. Thank you for taking the time to read and comment on our manuscript titled: ***Genetic association of microRNA-146a polymorphisms with the severity of coronary artery lesions in acute myocardial infarction****.* We have prepared the manuscript according to your comments.

Our responses are below:

**Major**

**Point 1:** Previous unmentioned study has demonstrated that patients with CAD carrying the miR‑146a rs2910164 C allele show lower expression of miR‑146a and increased expression of its target gene, NOS1 (Mol Med Rep. 2018 Jul;18(1):603-609.). The authors should discuss their results in the light of these previous evidence. Moreover, additional discussion should be provided on the relationship between miRNA-146a and genetic variance of NOS1 and endothelin which are mainly involved in endothelial dysfunction (J Transl Med. 2013 Sep 25:11:227.).

**Response 1:** We sincerely appreciate the editor’s valuable comments. We have supplemented the manuscript with additional evidence regarding the impact of the rs2910164 polymorphism on gene expression, as well as the reciprocal regulatory interaction between microRNA-146a and NOS1.

**Point 2:** The manuscript requires a careful revision of the style, grammar and flow.

**Response 2:** We have addressed and corrected these shortcomings in the revised manuscript.
