# Supplementary material for: Genetic association of microRNA-146a polymorphisms with the severity of coronary artery lesions in acute myocardial infarction
Source: PLoS One. 2025 Dec 26;20(12):e0339345. doi: 10.1371/journal.pone.0339345 (PMC12742729; doi:10.1371/journal.pone.0339345)
Supplement: Peer review history

# Peer review: Decision Letter 0

**DOI:** 10.1371/journal.pone.0339345.r001

**Authors:** Vincenzo Lionetti

6 Oct 2025

Dear Dr. Tran,

Thank you for submitting your manuscript to PLOS ONE. After careful consideration, we feel that it has merit but does not fully meet PLOS ONE’s publication criteria as it currently stands. Therefore, we invite you to submit a revised version of the manuscript that addresses the points raised during the review process.

==============================

**ACADEMIC EDITOR:**

Please submit your revised manuscript by Nov 20 2025 11:59PM. If you will need more time than this to complete your revisions, please reply to this message or contact the journal office at plosone@plos.org . When you're ready to submit your revision, log on to https://www.editorialmanager.com/pone/ and select the 'Submissions Needing Revision' folder to locate your manuscript file.

- A rebuttal letter that responds to each point raised by the academic editor and reviewer(s). You should upload this letter as a separate file labeled 'Response to Reviewers'.
- A marked-up copy of your manuscript that highlights changes made to the original version. You should upload this as a separate file labeled 'Revised Manuscript with Track Changes'.
- An unmarked version of your revised paper without tracked changes. You should upload this as a separate file labeled 'Manuscript'.

If you would like to make changes to your financial disclosure, please include your updated statement in your cover letter. Guidelines for resubmitting your figure files are available below the reviewer comments at the end of this letter.

If applicable, we recommend that you deposit your laboratory protocols in protocols.io to enhance the reproducibility of your results. Protocols.io assigns your protocol its own identifier (DOI) so that it can be cited independently in the future. For instructions see: https://journals.plos.org/plosone/s/submission-guidelines#loc-laboratory-protocols . Additionally, PLOS ONE offers an option for publishing peer-reviewed Lab Protocol articles, which describe protocols hosted on protocols.io. Read more information on sharing protocols at https://plos.org/protocols?utm_medium=editorial-email&utm_source=authorletters&utm_campaign=protocols .

We look forward to receiving your revised manuscript.

Kind regards,

Vincenzo Lionetti, M.D., PhD

Academic Editor

PLOS ONE

Journal Requirements:

When submitting your revision, we need you to address these additional requirements.

1. Please ensure that your manuscript meets PLOS ONE's style requirements, including those for file naming. The PLOS ONE style templates can be found at

https://journals.plos.org/plosone/s/file?id=wjVg/PLOSOne_formatting_sample_main_body.pdf and https://journals.plos.org/plosone/s/file?id=ba62/PLOSOne_formatting_sample_title_authors_affiliations.pdf

2. When completing the data availability statement of the submission form, you indicated that you will make your data available on acceptance. We strongly recommend all authors decide on a data sharing plan before acceptance, as the process can be lengthy and hold up publication timelines. Please note that, though access restrictions are acceptable now, your entire data will need to be made freely accessible if your manuscript is accepted for publication. This policy applies to all data except where public deposition would breach compliance with the protocol approved by your research ethics board. If you are unable to adhere to our open data policy, please kindly revise your statement to explain your reasoning and we will seek the editor's input on an exemption. Please be assured that, once you have provided your new statement, the assessment of your exemption will not hold up the peer review process.

3. Your ethics statement should only appear in the Methods section of your manuscript. If your ethics statement is written in any section besides the Methods, please delete it from any other section.

4. Please include captions for your Supporting Information files at the end of your manuscript, and update any in-text citations to match accordingly. Please see our Supporting Information guidelines for more information: http://journals.plos.org/plosone/s/supporting-information .

If the reviewer comments include a recommendation to cite specific previously published works, please review and evaluate these publications to determine whether they are relevant and should be cited. There is no requirement to cite these works unless the editor has indicated otherwise. 

[Note: HTML markup is below. Please do not edit.]

Reviewers' comments:

Reviewer's Responses to Questions

1. Is the manuscript technically sound, and do the data support the conclusions?

Reviewer #1: Partly

Reviewer #2: Yes

Reviewer #3: Yes

**********

2. Has the statistical analysis been performed appropriately and rigorously?

Reviewer #1: No

Reviewer #2: Yes

Reviewer #3: Yes

**********

3. Have the authors made all data underlying the findings in their manuscript fully available?

The PLOS Data policy

Reviewer #1: No

Reviewer #2: Yes

Reviewer #3: Yes

**********

4. Is the manuscript presented in an intelligible fashion and written in standard English?

Reviewer #1: Yes

Reviewer #2: Yes

Reviewer #3: Yes

**********

Reviewer #1: The study “Polymorphic characteristics of microRNA-146a gene at rs2431697, rs57095329, and rs2910164 and Its association with the severity of coronary artery lesions in patients with acute myocardial infarction” was performed by Ngo et al.

The paper showed the association between several polymorphism of microRNA-146a gene with acute myocardial infarction.

There were several unclear issues and limitations that need to be addressed:

- The title is too long and has grammar errors, please check.

- The introduction part is scanty. The authors should clarify the reasons why this gene was chosen to study, especially the connection between these polymorphisms and acute myocardial infarction. What are the roles of these SNPs in the development of acute myocardial infarction? Furthermore, the authors did not clearly present what the aims of this study are.

- The results were confused as the authors presented many comparisons. I suggest that the authors should clarify the aims of the study and focus on these aims while presenting the results.

- In table 3, why did the authors combine 2 genotypes in 1 group? Any specific reasons for that?

- I suggest that the authors should focus on univariate and multivariate logistic regression analyses for AMI (with and without disease) and severity of coronary results.

Reviewer #2: Dear/ Authers

The manusript idea is new- Title is long but accepted. Subjects and Methods: are well described , however I prefer to measure the expression of Mir 146 beside genotypings. Results: is clear but I prefer to evaluate Hardy-Weinberg equilibrium in groups, Measurment the Expression of mir 146 in cases will give a view on the effect of polymorphisms on mir 146 production.

Reviewer #3: The authors aim to investigate the characteristics of microRNA-146a gene polymorphisms (rs2431697, rs57095329, rs2910164) and their association with the severity of coronary artery lesions. The study is interesting, but there are a number of comments that should be addressed.

Major comments :

-Please elaborate why miRNA-146a was chosen as the vascular miRNA among the numerous documented miRNA markers associated with CVD risk. The study conclusions would be - strengthened by measuring a larger panel of miRNAs in a larger cohort.

-Provide a more thorough description of the SNPs and their potential impact on miRNA expression, as this information is currently lacking from the manuscript.

-Please introduce the aim of the study in the introduction section.

-The results section would be better if it were divided into subsections to make the message clearer to the reader. There are also too many tables; Tables 1 and 2 could be grouped together.

Minor comments:

-Include the missing p-values for the comparisons in both the abstract and the results section.

-The alleles and genotypes should be italicized.

**********

what does this mean? ). If published, this will include your full peer review and any attached files.

If you choose “no”, your identity will remain anonymous but your review may still be made public.

Do you want your identity to be public for this peer review? For information about this choice, including consent withdrawal, please see our Privacy Policy

Reviewer #1: No

Reviewer #2: No

Reviewer #3: No

**********

[NOTE: If reviewer comments were submitted as an attachment file, they will be attached to this email and accessible via the submission site. Please log into your account, locate the manuscript record, and check for the action link "View Attachments". If this link does not appear, there are no attachment files.]

While revising your submission, please upload your figure files to the Preflight Analysis and Conversion Engine (PACE) digital diagnostic tool, https://pacev2.apexcovantage.com/ . PACE helps ensure that figures meet PLOS requirements. To use PACE, you must first register as a user. Registration is free. Then, login and navigate to the UPLOAD tab, where you will find detailed instructions on how to use the tool. If you encounter any issues or have any questions when using PACE, please email PLOS at figures@plos.org . Please note that Supporting Information files do not need this step.

# Author comment: Author response to Decision Letter 1

**DOI:** 10.1371/journal.pone.0339345.r002

20 Nov 2025

Response to Reviewer 1 Comments

Dear Reviewers, we are happy to receive your comments. Thank you for taking the time to read and comment on our manuscript titled Genetic variations of microRNA-146a and their association with the severity of coronary artery lesions in patients with acute myocardial infarction. We have prepared the manuscript according to the your comments.

Our responses are below:

Major

Point 1: The title is too long and has grammar errors, please check.

Response 1: We have revised the title accordingly: Genetic variations of microRNA-146a and their association with the severity of coronary artery lesions in patients with acute myocardial infarction

Point 2: The introduction part is scanty. The authors should clarify the reasons why this gene was chosen to study, especially the connection between these polymorphisms and acute myocardial infarction. What are the roles of these SNPs in the development of acute myocardial infarction? Furthermore, the authors did not clearly present what the aims of this study are.

Response 2: We sincerely appreciate the reviewer’s insightful comments. We have added a description of the role of microRNA-146a to the Introduction and have also incorporated the study objectives into both the Abstract and the Introduction.

Point 3: The results were confused as the authors presented many comparisons. I suggest that the authors should clarify the aims of the study and focus on these aims while presenting the results.

Response 3: We have revised the multivariable analysis to place greater emphasis on evaluating the impact of risk factors and the rs2431697, rs57095329, and rs2910164 polymorphisms on the severity of coronary artery lesions. We sincerely appreciate your valuable feedback.

Point 4: In table 3, why did the authors combine 2 genotypes in 1 group? Any specific reasons for that?

Response 4: The combination of two genotypes in the analysis was intended to measure the effect of genotypes carrying the risk allele compared with those carrying no risk alleles. In addition, genotype-based analysis captures a full range of genetic inheritance models, including dominant, recessive, homozygous, and heterozygous patterns. While allele-based analysis only reflects differences in allele frequencies between cases and controls, genotype analysis allows for a direct evaluation of the genetic effect of a variant-for example, the dominant model (AA + Aa vs. aa), the recessive model (AA vs. Aa + aa), and assessment of Hardy-Weinberg equilibrium

Point 5: Suggest that the authors should focus on univariate and multivariate logistic regression analyses for AMI (with and without disease) and severity of coronary results.

Response 5: We sincerely appreciate the reviewer’s valuable comments. Our study aimed to evaluate the impact of microRNA-146a gene polymorphisms on the severity of coronary artery lesions in patients with acute myocardial infarction, rather than to assess their contribution to the risk of myocardial infarction onset. Nevertheless, we have further clarified both the univariable and multivariable models by incorporating clinical risk factors, biomarkers, and clinical scoring systems to better quantify their effects on the severity of coronary artery involvement.

Response to Reviewer 2 Comments

Major

Point 1: The manusript idea is new- Title is long but accepted. Subjects and Methods: are well described , however I prefer to measure the expression of Mir 146 beside genotypings. Results: is clear but I prefer to evaluate Hardy-Weinberg equilibrium in groups, Measurment the Expression of mir 146 in cases will give a view on the effect of polymorphisms on mir 146 production.

Response 1: We sincerely appreciate the reviewer’s insightful comments. Assessing the expression levels of microRNA-146a in addition to genotyping is indeed an excellent suggestion. However, due to limitations in our current resources, we were unable to perform this analysis in the present study. We will consider incorporating this important aspect in future research if feasible. The Hardy–Weinberg equilibrium assessment has now been added to the Results section of the revised manuscript.

Response to Reviewer 3 Comments

Major

Point 1: Please elaborate why miRNA-146a was chosen as the vascular miRNA among the numerous documented miRNA markers associated with CVD risk. The study conclusions would be - strengthened by measuring a larger panel of miRNAs in a larger cohort.

Response 1: We sincerely appreciate the reviewer’s valuable comments. Our study was conducted based on the premise that microRNA-146a is a circulating microRNA that plays a pivotal role in post-transcriptional gene regulation through its control of key inflammatory pathways, including the IL-1R/TLRs–NFκB axis, the Janus kinase (JAK)–STAT pathway, and fibrosis-related signaling. As microRNA-146a is involved in nearly all major regulatory pathways of the inflammatory response, alterations affecting this microRNA may influence inflammatory activity and thereby contribute to coronary artery injury. Supporting evidence shows that the rs2910164 polymorphism can alter gene expression, is associated with dysregulation of inflammatory cytokines, and correlates with the severity of coronary artery lesions

Point 2: Provide a more thorough description of the SNPs and their potential impact on miRNA expression, as this information is currently lacking from the manuscript.

Response 2: This represents a limitation of our study. Due to restricted resources, our investigation was limited to genotypic characterization and did not include an assessment of how these polymorphisms influence gene expression or circulating microRNA levels in plasma

Point 3: Please introduce the aim of the study in the introduction section.

Response 3: We have added the study objectives and expanded the Introduction accordingly

Point 4: The results section would be better if it were divided into subsections to make the message clearer to the reader. There are also too many tables; Tables 1 and 2 could be grouped together.

Response 4: Thank you very much for your comment. Table 2 is included in the subsection titled ‘Characteristics of the rs2431697, rs57095329, and rs2910164 microRNA-146a polymorphisms,’ which has been separated from the general characteristics section. The intention of this structure is to emphasize that this subsection focuses specifically on describing the genetic polymorphisms and evaluating their effects on clinical risk factors

Minor

Point 5: Include the missing p-values for the comparisons in both the abstract and the results section. The alleles and genotypes should be italicized.

Response 5: Thank you very much. We have incorporated the requested revisions into the manuscript.

Attachment Submitted filename: Respond Reviewer.docx

# Peer review: Decision Letter 1

**DOI:** 10.1371/journal.pone.0339345.r003

**Authors:** Vincenzo Lionetti

30 Nov 2025

Dear Dr. Tran,

Thank you for submitting your manuscript to PLOS ONE. After careful consideration, we feel that it has merit but does not fully meet PLOS ONE’s publication criteria as it currently stands. Therefore, we invite you to submit a revised version of the manuscript that addresses the points raised during the review process.

==============================

**ACADEMIC EDITOR: ** Further discussion should be provided to strenghten relationship with occurrence of endothelian dysfunction. Moreover, a comprehensive editing of the text is required.

==============================

Please submit your revised manuscript by Jan 14 2026 11:59PM. If you will need more time than this to complete your revisions, please reply to this message or contact the journal office at plosone@plos.org . When you're ready to submit your revision, log on to https://www.editorialmanager.com/pone/ and select the 'Submissions Needing Revision' folder to locate your manuscript file.

- A rebuttal letter that responds to each point raised by the academic editor and reviewer(s). You should upload this letter as a separate file labeled 'Response to Reviewers'.
- A marked-up copy of your manuscript that highlights changes made to the original version. You should upload this as a separate file labeled 'Revised Manuscript with Track Changes'.
- An unmarked version of your revised paper without tracked changes. You should upload this as a separate file labeled 'Manuscript'.

If applicable, we recommend that you deposit your laboratory protocols in protocols.io to enhance the reproducibility of your results. Protocols.io assigns your protocol its own identifier (DOI) so that it can be cited independently in the future. For instructions see: https://journals.plos.org/plosone/s/submission-guidelines#loc-laboratory-protocols . Additionally, PLOS ONE offers an option for publishing peer-reviewed Lab Protocol articles, which describe protocols hosted on protocols.io. Read more information on sharing protocols at https://plos.org/protocols?utm_medium=editorial-email&utm_source=authorletters&utm_campaign=protocols .

We look forward to receiving your revised manuscript.

Kind regards,

Vincenzo Lionetti, M.D., PhD

Academic Editor

PLOS ONE

Journal Requirements:

1. If the reviewer comments include a recommendation to cite specific previously published works, please review and evaluate these publications to determine whether they are relevant and should be cited. There is no requirement to cite these works unless the editor has indicated otherwise. 

2. Please review your reference list to ensure that it is complete and correct. If you have cited papers that have been retracted, please include the rationale for doing so in the manuscript text, or remove these references and replace them with relevant current references. Any changes to the reference list should be mentioned in the rebuttal letter that accompanies your revised manuscript. If you need to cite a retracted article, indicate the article’s retracted status in the References list and also include a citation and full reference for the retraction notice.

Additional Editor Comments:

Major issues

1) Previous unmentioned study has demonstrated that patients with CAD carrying the miR‑146a rs2910164 C allele show lower expression of miR‑146a and increased expression of its target gene, NOS1 (Mol Med Rep. 2018 Jul;18(1):603-609.). The authors should discuss their results in the light of these previous evidence. Moreover, additional discussion should be provided on the relationship between miRNA-146a and genetic variance of NOS1 and endothelin which are mainly involved in endothelial dysfunction (J Transl Med. 2013 Sep 25:11:227.).

2) The manuscript requires a careful revision of the style, grammar and flow.

[Note: HTML markup is below. Please do not edit.]

Reviewers' comments:

Reviewer's Responses to Questions

**Comments to the Author**

Reviewer #1: All comments have been addressed

Reviewer #3: All comments have been addressed

**********

2. Is the manuscript technically sound, and do the data support the conclusions?

Reviewer #1: Yes

Reviewer #3: Yes

**********

3. Has the statistical analysis been performed appropriately and rigorously?

Reviewer #1: Yes

Reviewer #3: Yes

**********

4. Have the authors made all data underlying the findings in their manuscript fully available?

The PLOS Data policy

Reviewer #1: Yes

Reviewer #3: Yes

**********

5. Is the manuscript presented in an intelligible fashion and written in standard English?

Reviewer #1: Yes

Reviewer #3: Yes

**********

Reviewer #1: All my comments were addressed, I don't have any further comments. Congratulate the authors for the manuscript

Reviewer #3: The authors have adequately addressed all comments made by the reviewer. No additional modifications are required.

**********

what does this mean? ). If published, this will include your full peer review and any attached files.

If you choose “no”, your identity will remain anonymous but your review may still be made public.

**Do you want your identity to be public for this peer review?** For information about this choice, including consent withdrawal, please see our Privacy Policy

Reviewer #1: No

Reviewer #3: No

**********

[NOTE: If reviewer comments were submitted as an attachment file, they will be attached to this email and accessible via the submission site. Please log into your account, locate the manuscript record, and check for the action link "View Attachments". If this link does not appear, there are no attachment files.]

To ensure your figures meet our technical requirements, please review our figure guidelines: https://journals.plos.org/plosone/s/figures 

You may also use PLOS’s free figure tool, NAAS, to help you prepare publication quality figures: https://journals.plos.org/plosone/s/figures#loc-tools-for-figure-preparation. 

NAAS will assess whether your figures meet our technical requirements by comparing each figure against our figure specifications.

# Author comment: Author response to Decision Letter 2

**DOI:** 10.1371/journal.pone.0339345.r004

4 Dec 2025

Dear Editor, we are happy to receive your comments. Thank you for taking the time to read and comment on our manuscript. We have prepared the manuscript according to your comments.

Our responses are below:

Major

Point 1: Previous unmentioned study has demonstrated that patients with CAD carrying the miR‑146a rs2910164 C allele show lower expression of miR‑146a and increased expression of its target gene, NOS1 (Mol Med Rep. 2018 Jul;18(1):603-609.). The authors should discuss their results in the light of these previous evidence. Moreover, additional discussion should be provided on the relationship between miRNA-146a and genetic variance of NOS1 and endothelin which are mainly involved in endothelial dysfunction (J Transl Med. 2013 Sep 25:11:227.).

Response 1: We sincerely appreciate the editor’s valuable comments. We have supplemented the manuscript with additional evidence regarding the impact of the rs2910164 polymorphism on gene expression, as well as the reciprocal regulatory interaction between microRNA-146a and NOS1.

Point 2: The manuscript requires a careful revision of the style, grammar and flow.

Response 2: We have addressed and corrected these shortcomings in the revised manuscript.

Attachment Submitted filename: Respond Editor.docx

# Editor report: Decision Letter 2

**DOI:** 10.1371/journal.pone.0339345.r005

**Authors:** Vincenzo Lionetti

8 Dec 2025

Genetic association of microRNA-146a polymorphisms with the severity of coronary artery lesions in acute myocardial infarction

PONE-D-25-40705R2

Dear Dr. Tran,

We’re pleased to inform you that your manuscript has been judged scientifically suitable for publication and will be formally accepted for publication once it meets all outstanding technical requirements.

Within one week, you’ll receive an e-mail detailing the required amendments. When these have been addressed, you’ll receive a formal acceptance letter and your manuscript will be scheduled for publication.

An invoice will be generated when your article is formally accepted. Please note, if your institution has a publishing partnership with PLOS and your article meets the relevant criteria, all or part of your publication costs will be covered. Please make sure your user information is up-to-date by logging into Editorial Manager at Editorial Manager®  and clicking the ‘Update My Information' link at the top of the page. For questions related to billing, please contact billing support .

If your institution or institutions have a press office, please notify them about your upcoming paper to help maximize its impact. If they’ll be preparing press materials, please inform our press team as soon as possible -- no later than 48 hours after receiving the formal acceptance. Your manuscript will remain under strict press embargo until 2 pm Eastern Time on the date of publication. For more information, please contact onepress@plos.org.

Kind regards,

Vincenzo Lionetti, M.D., PhD

Academic Editor

PLOS One

Additional Editor Comments (optional):

Reviewers' comments:

# Editor report: Acceptance letter

**DOI:** 10.1371/journal.pone.0339345.r006

**Authors:** Vincenzo Lionetti

PONE-D-25-40705R2

PLOS One

Dear Dr. Tran,

I'm pleased to inform you that your manuscript has been deemed suitable for publication in PLOS One. Congratulations! Your manuscript is now being handed over to our production team.

At this stage, our production department will prepare your paper for publication. This includes ensuring the following:

* All references, tables, and figures are properly cited

* All relevant supporting information is included in the manuscript submission,

* There are no issues that prevent the paper from being properly typeset

You will receive further instructions from the production team, including instructions on how to review your proof when it is ready. Please keep in mind that we are working through a large volume of accepted articles, so please give us a few days to review your paper and let you know the next and final steps.

Lastly, if your institution or institutions have a press office, please let them know about your upcoming paper now to help maximize its impact. If they'll be preparing press materials, please inform our press team within the next 48 hours. Your manuscript will remain under strict press embargo until 2 pm Eastern Time on the date of publication. For more information, please contact onepress@plos.org.

You will receive an invoice from PLOS for your publication fee after your manuscript has reached the completed accept phase. If you receive an email requesting payment before acceptance or for any other service, this may be a phishing scheme. Learn how to identify phishing emails and protect your accounts at https://explore.plos.org/phishing.

If we can help with anything else, please email us at customercare@plos.org.

Thank you for submitting your work to PLOS ONE and supporting open access.

Kind regards,

PLOS ONE Editorial Office Staff

on behalf of

Prof. Vincenzo Lionetti

Academic Editor

PLOS One
